# Multivessel coronary artery ectasia and severe calcification in a patient with pheochromocytoma: a case report

**DOI:** 10.7555/JBR.32.20170047

**Published:** 2018-05-28

**Authors:** Daokuo Yao, Xiangyu Gao, Huiqiang Zhao, Hui Chen, Lexin Wang

**Affiliations:** 1. Department of Cardiology, Beijing Friendship Hospital, Capital Medical University, Beijing 100050, China; 2. Department of Cardiology, Liaocheng People’s Hospital, Liaocheng, Shandong 252000, China; 3. School of Biomedical Sciences, Charles Sturt University, Wagga Wagga, NSW 2650, Australia.

**Keywords:** myocardial infarction, coronary artery ectasia, percutaneous coronary intervention, pheochromocytoma, hypertension

## Abstract

Multivessel coronary artery ectasia with severe calcification is rare among patients with coronary artery disease. A 74-year-old Chinese woman suffered from acute myocardial infarction on a background of 50 years of poorly controlled hypertension secondary to pheochromocytoma, which was surgically removed in June 2012 prior to the presentation. Coronary angiography revealed total occlusion of the proximal left anterior descending artery, and multiple ectasias with severe calcification in the left main, circumflex and right coronary artery. After an aspiration thrombectomy and balloon angioplasty, grade 3 coronary flow was restored in the left descending coronary artery. No cardiac events were found in the 12-month follow-up. We conclude that multivessel coronary artery ectasia and severe calcification may be present in patients with a long-standing history of hypertension secondary to pheochromocytoma.

## Introduction

Coronary artery ectasia is uncommon among patients with coronary artery disease. It is mainly diagnosed incidentally when coronary angiograms are performed to investigate various chest pains or complaints^[1–3]^. Multivessel coronary artery ectasia, especially with severe calcification, is very rare. There have been only very few cases of acute myocardial infarction (AMI) associated with coronary artery ectasia in previous reports^[2,4]^. In this study, we present a case with AMI and multivessel coronary artery ectasia on a background of long standing hypertension secondary to pheochromocytoma.

## Case report

This study was approved by the human ethics committee of our hospital. A 74-year-old Chinese woman presented with two hours of central chest pain accompanied by diaphoresis. She has a history of hypertension for 50 years with sporadic medication therapy. Five years ago, a pheochromocytoma (3.8 cm× 3.0 cm×2.6 cm, 20 g) was found on the right adrenal gland with an increased plasma level of noradrenaline (2.041 nmol/L, normal reference range, 0.104–0.548 nmol/L). The pheochromocytoma was successfully resected and the blood pressure returned to the normal range following the surgery. There was no family history of pheochromocytoma or coronary artery disease. She was a non-smoker and did not drink alcohol.

On physical examination, her blood pressure was 136/78 mmHg and heart rate was 86 beats/minute. Heart sounds were normal and no murmur or pericardial rub was audible. Electrocardiography showed ST segment elevations in leads V1–V5. Cardiac troponin I was mildly elevated to 0.086 mg/mL (normal reference range<0.030 mg/mL). She was diagnosed with acute anterior myocardial infarction.

**Fig.1:**
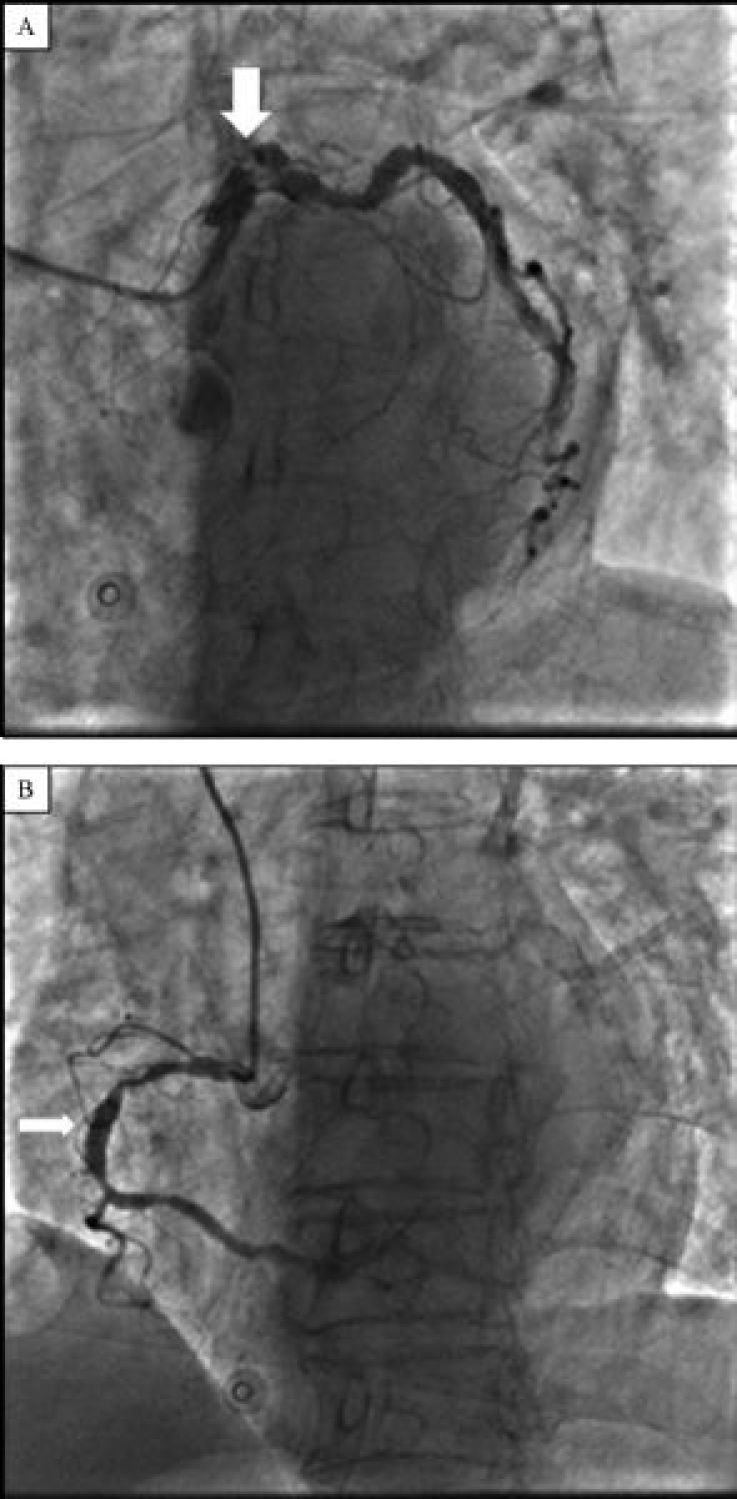
**Left and right coronary angiographic images.** A: Total occlusion of the proximal left anterior descending artery with multiple ectasias and severe calcification in the left main (arrow) and circumflex coronary artery. B: Right coronary angiographic image. Multiple ectasias and severe calcification (arrow).

**Fig.2:**
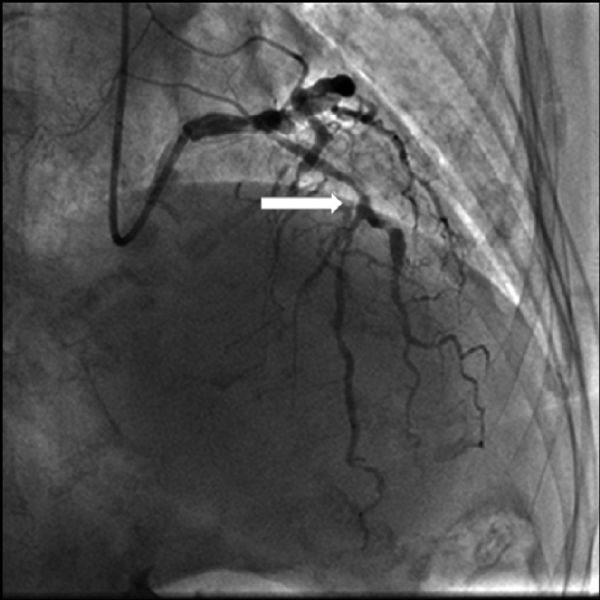
**After aspiration thrombectomy and balloon angioplasty.** The antegrade blood flow of the left anterior descending artery was restored (arrow).

After a load dose of aspirin 300 mg and clopidogrel 600 mg, the patient was transferred to the catheterization laboratory for emergency coronary angiography, which revealed a total occlusion of the proximal left anterior descending artery (LAD), and multiple ectasias and severe calcification in the left main, circumflex and right coronary artery ( ***Fig. 1***). Percutaneous coronary intervention (PCI) was attempted. A guide wire was passed through the occluded lesion to the distal of LAD, and the thrombus was aspirated by a ZEEK aspiration catheter. The antegrade flow of LAD was restored to TIMI (thrombolysis in myocardial infarction) 2. Stenosis was present in the proximal of LAD and the first diagonal artery, and a pre-dilatation was done with a 2.5/15 mm Sprinter balloon for 60 seconds. The antegrade flow of LAD was improved to TIMI 3 after the dilation ( ***Fig. 2***).

Chest pain was relieved after the emergency PCI and the patient was admitted to the coronary care unit. She was treated with subcutaneous enoxaparin, oral aspirin, clopidogrel, and rosuvastatin in the following days, and discharged seven days after the PCI. She remained asymptomatic during the follow-up for 12 months.

The patient has provided informed consent for the publication of the case. Patient data were anonymized in this report.

## Discussion

Coronary artery ectasia is defined as the presence of an enlarged segment greater than 1.5 times the diameter of the adjacent normal coronary artery^[1–2]^. Most coronary artery ectasia is asymptomatic and is found occasionally by angiography. The incidence of coronary artery ectasia is 1.5%–5.0% among patients undergoing coronary angiography^[1,3]^. About 75% patients with coronary artery ectasia have isolated artery involvement, and multivessel coronary artery ectasia is very rare^[3]^.

Atherosclerosis is the most common cause of coronary artery ectasia, accounting for about 50% of the reported cases^[1]^. Kawasaki disease is the most common non-atherosclerotic cause for coronary artery ectasia. Other uncommon causes of coronary artery ectasia include infection, inflammatory or connective tissue disease, such as scleroderma^[1]^. Iatrogenic causes, such as deep vessel injury by balloon angiography or stenting, have recently been reported^[1]^. In our case, the long standing and poorly controlled hypertension secondary to pheochromocytoma may have played a role in the pathogenesis of multivessel coronary artery ectasia and calcification. Pheochromocytoma is associated with many cardiac complications, such as cardiomyopathy, arrhythmia, hypertension, or acute coronary syndrome, but there has been no previous report on the association between pheochromocytoma and multivessel coronary artery ectasia. Pheochromocytoma is associated with coronary artery fibromuscular dysplasia^[5]^. However, whether coronary artery dysplasia increases the risk of ectasia is unclear.

Coronary artery ectasia can be complicated by thrombosis, embolism or rupture of the coronary segments involved^[2]^. In patients with coronary artery ectasia, myocardial infarction can occur from *in situ* thrombosis or from embolization^[2]^. Multivessel coronary artery ectasia, although very rare, are mainly seen in patients with myocardial infarction or angina pectoris undergoing coronary angiography^[6–8]^.

There has been a lack of consensus on the optimal therapy for coronary artery ectasia. Medical therapy with antiplatelet agents might reduce thrombosis formation or embolization. In patients with acute coronary syndrome and with significant thrombosis formation, aspiration thrombectomy is safe and effective^[9]^. Polytetrafluoroethylene-covered stents may be used for large or bulky coronary ectasia lesions^[10]^. The role of drug-eluting stents in the treatment of coronary ectasia is uncertain, as a number of ectasia cases were reported after implantation of drug-eluting stents, which may case coronary artery remodeling and progressive luminal dilation^[10]^. Coronary bypass surgery may be used only for very large aneurysms involving many segments. Our patient was treated with aspiration thrombectomy and coronary balloon angioplasty, followed by medication therapies. At the end of 12-month follow up, the patient remained asymptomatic.

## Conclusions

Multivessel coronary artery ectasia is rare and can be presented initially as AMI. Its etiology is unclear but arthrosclerosis and long standing hypertension seem to play a critical role. The optimal treatment strategies for multivessel coronary artery ectasia are yet to be defined, but polytetrafluoroethylene-covered stents seem to be effective and safe in selected patients. Coronary artery bypass grafting is the ultimate therapy for severe multivessel coronary artery ectasia.
